# Complex endovascular repair of type B aortic dissection and predicting left arm ischemia: a case report

**DOI:** 10.1186/s13256-021-02772-y

**Published:** 2021-04-15

**Authors:** Kevin G Kim, Anthony N Grieff, Saum Rahimi

**Affiliations:** Division of Vascular Surgery and Endovascular Therapy, Rutgers Robert Wood Johnson School of Medicine, One Robert Wood Johnson Place, MEB 541, New Brunswick, NJ 08901 USA

**Keywords:** Subclavian artery coverage, TEVAR, Type B dissection, Thoracic aortic aneurysm, Zone 2 coverage

## Abstract

**Background:**

Thoracic endovascular aortic repair (TEVAR) is the gold standard for surgical management of descending thoracic aortic pathology. Depending on the anatomy, TEVAR often requires deployment across the origin of the left subclavian artery (LSA) to obtain a proximal seal, thus potentially compromising perfusion to the left upper extremity (LUE). However, in most patients this is generally well tolerated without revascularization due to collateralization from the left vertebral artery (LVA).

**Case presentation:**

We present a complex 59-year-old Caucasian patient case of TEVAR with a history of prior arch debranching and intraoperative LSA coverage requiring subsequent LSA embolization and emergency take-back for left carotid–subclavian bypass.

**Conclusion:**

The purpose of this case report is to highlight an often overlooked anatomic LVA variant and an atypical, delayed presentation of acute LUE limb ischemia.

## Background

Thoracic endovascular aortic repair (TEVAR) is the gold-standard surgical management of thoracic aortic pathology and is associated with superior 30-day survival rates compared with traditional open repair [1]. It is not uncommon for grafts to require deployment across the origin of the great vessels to obtain proximal seal, thus potentially compromising upper extremity/cerebrovascular perfusion. For this reason, hybrid approaches have emerged for preemptively bypassing and debranching the innominate artery (IA) and left common carotid artery (LCCA) from the proximal aortic arch [2, 3]. Revascularization of the left subclavian artery (LSA) is difficult with standard sternotomy, requiring additional thoracotomy; however, symptomatic ischemia from LSA coverage has been reported to occur in only a modest 6–10% of patients and is often sacrificed with impunity given coverage rates between 10–50% [4–6]. This is because of multiple collaterals beyond the LSA origin, notably retrograde flow from the left vertebral artery (LVA), the occipital branch of the external carotid artery, and the superior thyroid artery [6]. We present a complex case of TEVAR with a history of prior arch debranching and intraoperative LSA coverage requiring subsequent LSA embolization and emergency take-back for left carotid–subclavian bypass. This case report highlights an often overlooked anatomic LVA variant and an atypical, delayed presentation of acute left upper extremity (LUE) limb ischemia.

## Case presentation

**Fig. 1:**
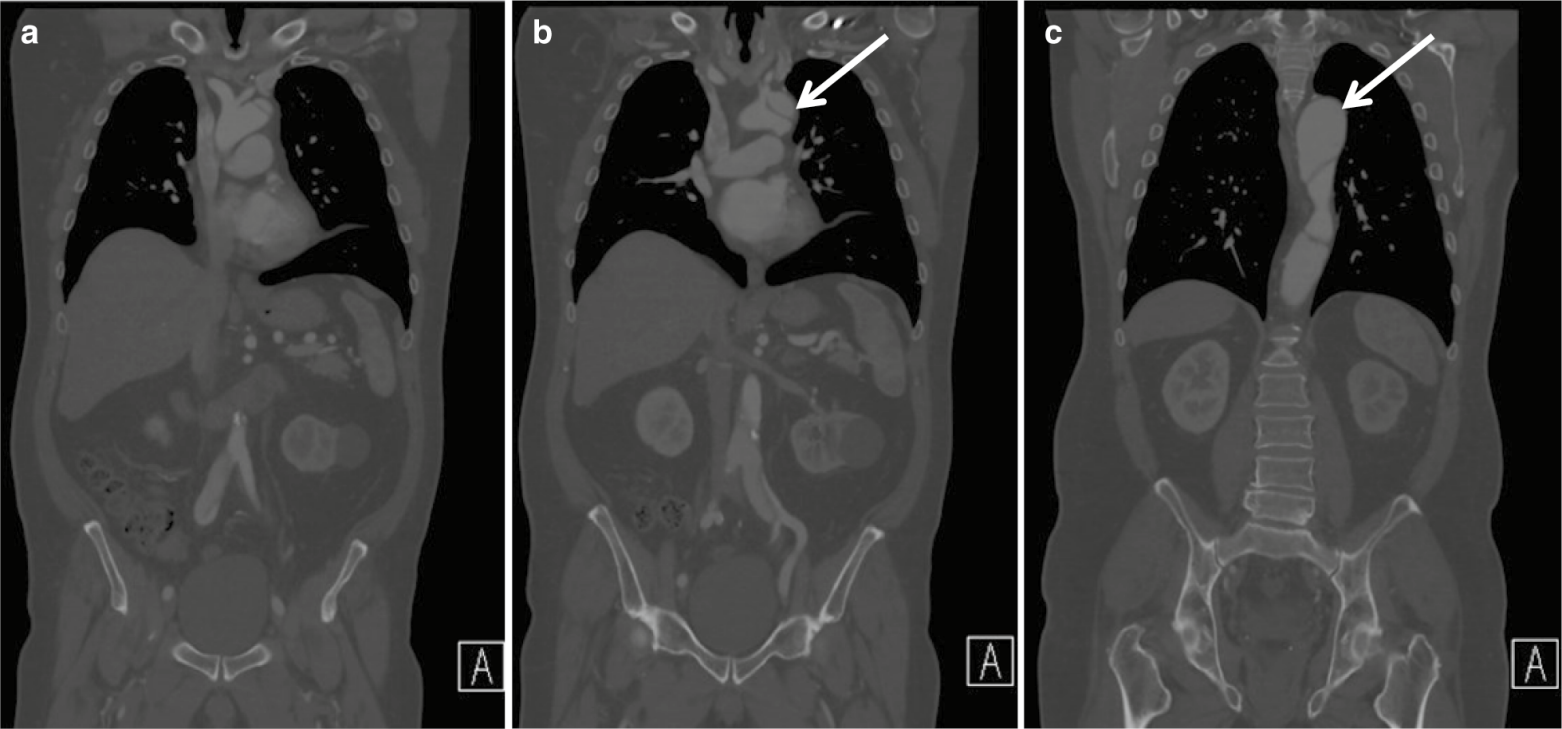
Surveillance computed tomography angiography coronal cuts from anterior to posterior (**a**–**c**, respectively). Arrows correspond to a chronic type B dissection involving the ostium of the left subclavian artery with aneurysmal degeneration to 5.3 cm. Distally, there is extension into the iliac bifurcation. There are multiple thoracic fenestrations, with all visceral vessels off the true lumen

A 59-year-old Caucasian patient at the time of presentation had been followed for several years by cardiac surgery for a history of a chronic type B aortic dissection, of hypertensive etiology, involving the ostium of the LSA and extending to the iliac bifurcation with multiple fenestrations. He was referred to vascular surgery due to progressive degeneration of a thoracic aortic aneurysm involving the origin of the LSA, which had increased from 4.5 cm to 5.3 cm over a 1-year surveillance period (Fig. 1).

**Fig. 2:**
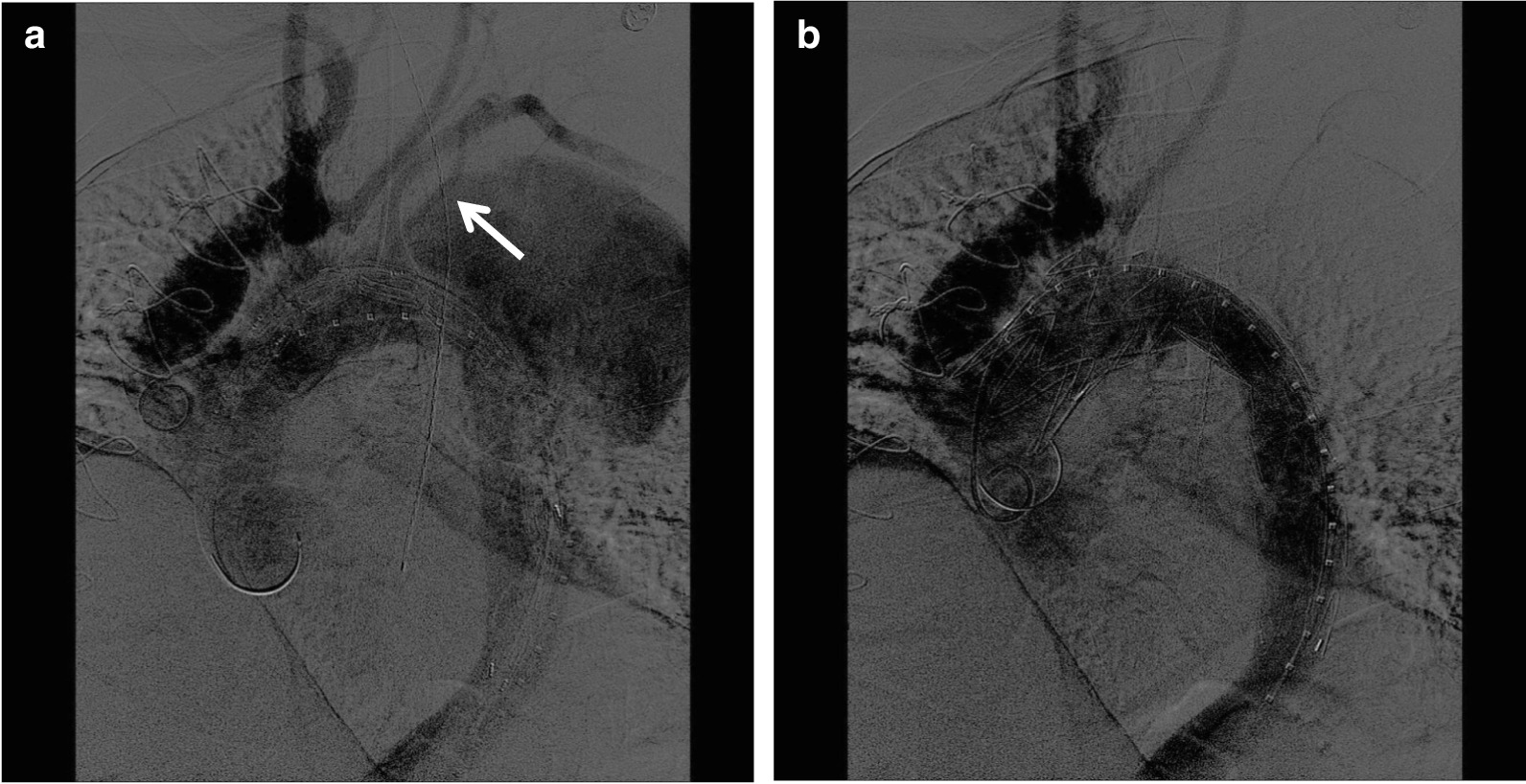
Thoracic stenting. **a** Prior to stent deployment, there is brisk filling of the neo-origins of the innominate and right carotid arteries after debranching. As indicated by the arrow, the left vertebral artery takes its origin directly from the aortic arch. The thoracic aneurysm had increased to a maximal diameter of 9 cm. **b** There is complete exclusion of the aneurysm after stent graft deployment. The innominate and right carotid arteries remain patent. There is no perfusion to the left subclavian artery or vertebral artery

Given the patient’s pathology, successful TEVAR would require covering the patient’s common ostium of the IA and LCCA. Therefore, a hybrid approach in conjunction with cardiac surgery was selected. The patient first underwent an open debranching of the IA and LCCA with bypass to the proximal aorta. Approximately 1 year later on repeat imaging, the patient’s aneurysm had rapidly expanded to approximately 9 cm, prompting urgent coverage with a 36 × 200 mm Bolton thoracic stent graft just distal to the bypass, resulting in coverage of the LSA origin to exclude the aneurysm (Fig. 2). On angiography, we noted that the patient had an aberrant origin of the LVA off the arch that required coverage, placing the patient at risk for spinal or LUE ischemia; however the patient had a palpable radial pulse after coverage and no complaints during subsequent recovery. The patient was discharged home on postoperative day 2 without complications.

**Fig. 3:**
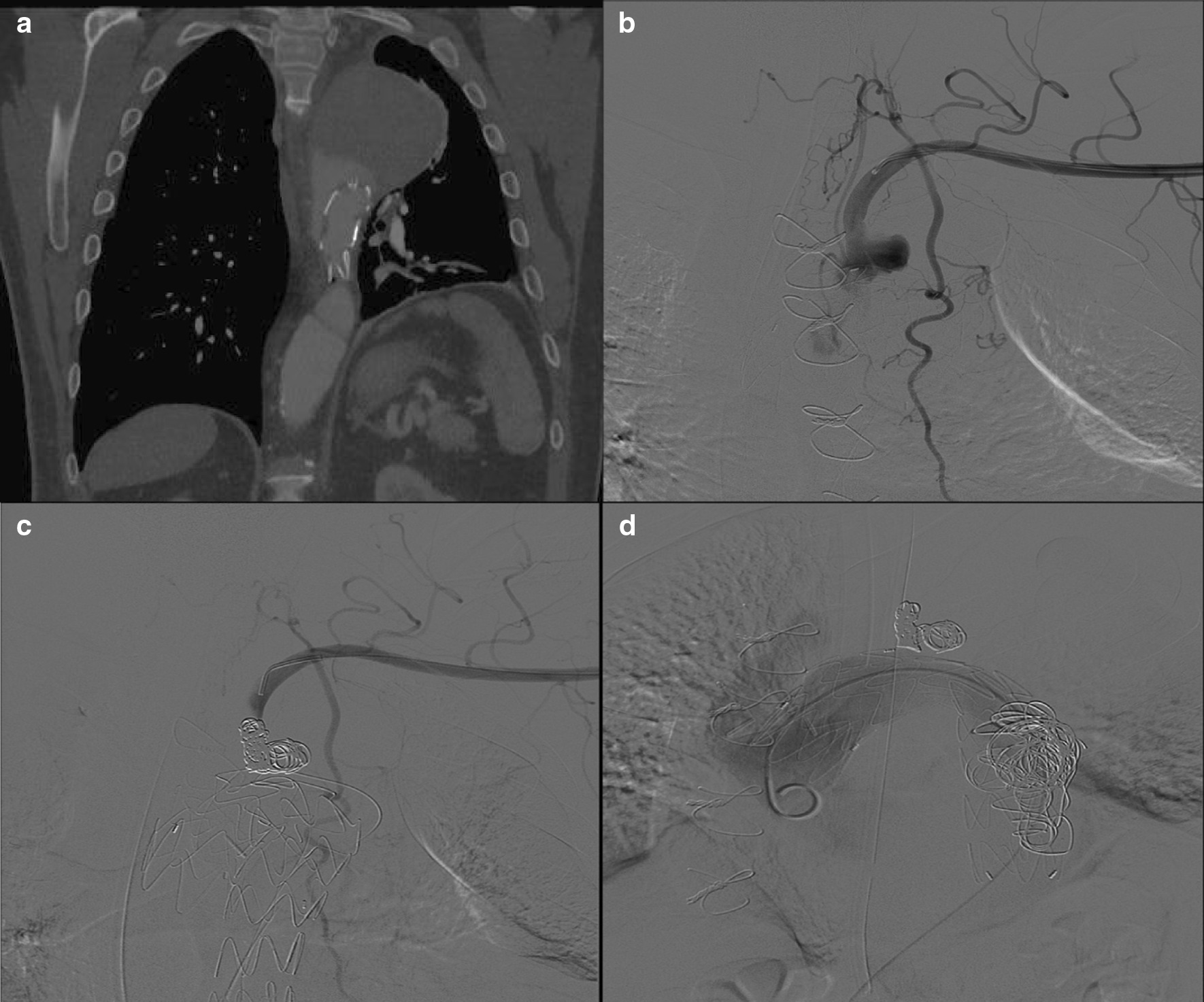
**a** Post-thoracic endovascular aortic repair (TEVAR) computed tomography angiography demonstrating an endoleak around the TEVAR, likely type II from multiple thoracic perforators and left subclavian artery (LSA). **b** Angiogram from left brachial catheter demonstrating type II endoleak from the LSA filling the aneurysm sac and multiple collaterals. **c** Coil embolization of the LSA with subsequent absent filling of the aneurysm sac. **d** Angiogram at the level of the TEVAR after coil embolization of the false lumen with no evidence of endoleak

Ten days post-TEVAR, the patient presented to the emergency department with acute worsening chest pain radiating to the neck and back. On computed tomography angiography (CTA) he was found to have a newly discovered type II endoleak from the covered LSA in addition to several thoracic perforators and a possible type Ib endoleak from the distal false lumen (Fig. 3a, b). Given his symptoms, the patient was urgently taken back to the operating room for revision, in which the proximal LSA was coil-embolized via a left brachial artery cutdown in addition to false lumen coil embolization via a retrograde femoral approach (Fig. 3c, d).

Immediately following surgery, the patient began complaining of LUE pain and numbness, with clinical concern for acute ischemia. The patient was taken back to the operating room and underwent urgent left carotid–subclavian bypass to restore LUE perfusion. The remaining hospital course was uncomplicated, and the patient was eventually discharged with no complaints of chest pain or further evidence of acute limb ischemia.

## Discussion and conclusions

With the expanding use of TEVAR for aortic arch pathology, it is increasingly important to be aware of LUE ischemia and atypical patient anatomy. In the recent literature, the inherent risks of LSA coverage without preoperative revascularization were found to include LUE ischemia (6–10%), stroke (2.6%), spinal cord ischemia (4%), endoleak (1.2%), and myocardial infarction [4–6]. In our case, the patient developed acute LUE ischemia due to an aberrant LVA which was clinically silent due to persistent LSA perfusion from the patent false lumen. This is noteworthy, because the patient had already tolerated LSA coverage without symptoms, and there was low clinical suspicion for postoperative ischemia, as would have been the case during the initial coverage.

Although fairly uncommon, missed sequelae of LSA coverage can be devastating, and 2010 recommendations from the Society for Vascular Surgery (SVS) suggest preemptive revascularization for any elective TEVAR that results in coverage of the LSA [2]; however, these recommendations were based on low-grade evidence derived from observational studies, case series, and expert opinions [2]. Despite official Society recommendations, only modest rates (0–73%) of preoperative revascularization are documented in the literature, which may be explained by the lack of high-grade evidence to support LSA revascularization during TEVAR procedures [6]. Therefore, many authors suggest that it is not unreasonable to cover the LSA despite the known risks of malperfusion to the brain, spinal cord, and LUE, as the aortic pathology is more urgent [3, 8].

A recent study evaluated an intraoperative protocol for LSA revascularization using measurements from bilateral radial artery catheters to assess left radial artery pulsatility, systolic pressure differences between left and right upper extremities, and LUE pulse oximetry [9, 10] This approach yielded a revascularization rate of 2.6%, compared to 6.5% in the literature, with no adverse events [6, 9]. Further evaluation of a selective revascularization strategy optimized to diminish postoperative revascularization represents an important area of future research, as a recent study has suggested that post-TEVAR revascularization is associated with worse clinical outcomes [11].

The method of choice for revascularization is subclavian transposition (SCT) or carotid–subclavian bypass, with patency rates of 99% and 86%, respectively. SCT is the preferred method, with its superior patency and exclusion of complications associated with grafts (infection, kinks, aneurysms). Revascularization does carry its own inherent risks, which the literature reports as nerve injury in 11.2%, stroke in 4.4%, lymphatic leak in 2.4%, and hematoma in 0.9% of SCT cases. Despite these risks, revascularization with either method before or during TEVAR has been shown to reduce the risk of LUE ischemia to 0% [12]. Sobocinski [13] further confirmed a statistically significant reduction in ischemic symptoms associated with LSA revascularization.

In this case, we suspect that aberrant LVA anatomy was the likely cause of our patient’s LUE ischemia. While aberrant LVA of aortic origin is uncommon, and seen in only 2.9% of patients, the origin is usually (86.3%) between the LCCA and LSA [14, 15, 16]. Our patient also had a bovine arch (13.6% of population), but this is not indicative of isolated LVA anatomy, as this dual aberrancy occurs in only 0.4% of patients [15]. It should be noted that beyond aberrant LVA, other anatomic variants known to increase LUE risk with LSA coverage include incomplete circle of Willis, right vertebral artery insufficiency, aberrant right subclavian artery, aberrant CCA and right-sided aortic arch [6, 7, 15]. Although an aberrant LVA is uncommon, it was recently found that the incidence of isolated LVA in thoracic aortic disease may be higher than that in the general population (6.3% vs. 3.4%, *p* < 0.001), suggesting that aberrant LVA could be a novel marker for pathology [17].

In conclusion, this case highlights an unusual case of delayed LUE ischemia after LSA coverage in the setting of aberrant LVA anatomy. It is important to carefully evaluate patient anatomy when planning TEVAR and to maintain vigilance in detecting LUE malperfusion when LSA coverage has occurred.

## Data Availability

Not applicable.
